# SFPQ subcellular mislocalisation entails altered cancer cell function

**DOI:** 10.1002/ctm2.70431

**Published:** 2025-08-06

**Authors:** Libang Yang, Adam Gilbertsen, Blake Jacobson, Robert Kratzke, Yingming Li, Bo Sun, Sabine Karam, Scott M. Dehm, Craig A. Henke

**Affiliations:** ^1^ Department of Medicine School of Medicine University of Minnesota Minneapolis Minnesota USA; ^2^ Department of Hematology Oncology and Transplantation, School of Medicine, University of Minnesota Minneapolis Minnesota USA; ^3^ Masonic Cancer Center, Department of Medicine, University of Minnesota Minneapolis Minnesota USA; ^4^ Division of Gastro, Hepatology, Nutrition, Department of Medicine University of Minnesota Minneapolis Minnesota USA; ^5^ Division of Nephrology, Department of Medicine University of Minnesota Minneapolis Minnesota USA

1

Dear Editor,

This study demonstrates that splicing factor proline and glutamine rich (SFPQ) is present on the cell surface in cancer cells, where it interacts with S100A4 and so forth proteins to alter cell function when SFPQ is a nuclear protein normally. Protein subcellular mislocalisation can alter cell function in cancer development, metastasis or drug resistance and may serve as a therapy target.^1^ Overexpression of SFPQ is essential for the malignancy of cancer cells.^2^, ^3^, ^4^ While SFPQ normally resides in the nucleus, it can be found in other subcellular locations in lung cancer cells.^5^ We further explore if SFPQ appears on the cell surface and its influence on cancer cells.

SFPQ is more highly stained in the cancer nucleus and cytoplasmic region than the adjacent normal area in immunohistochemistry (IHC) stain in multiple cancer tissues (Figure 1A‒J). The cytoplasmic staining was 3.8‒8.6 times greater than that of the control region and nuclear staining was also more prominent (3.6‒5.6 times more than the control regions in stain density quantification analysis; Figure 1B,D,F,H,I). SFPQ is present outside of the cell nucleus in solid cancers in most cases but not in control tissues. To observe the SFPQ distribution in subcellular organelles, the cell membrane, cytoplasm and nucleus were isolated and used in a Western blot to observe the SFPQ level. With subcellular fractions from normal and cancer cell lines, we found that SFPQ was present in all of the cell membranes, cytoplasm and nuclear fractions from cancer cells but only in nuclear fractions from non‐cancer cell lines (Figure 1K‒M). We then used immunofluorescence staining to determine whether SFPQ is present on the cell surface of solid cancer cell lines. We fixed the cells with paraformaldehyde (which fixes the cells but does not penetrate the cell membrane) and stained them with an SFPQ antibody. Most of the cancer cells were positive for SFPQ, which suggested that SFPQ was present on the cell surface (Figure 1N). Internalisation assay with pHrodo‐labelled SFPQ antibody and lung cancer cell H661 was positive, suggesting SFPQ is a cell surface protein^6^ (Figure 1O). Additionally, we found SFPQ and the cell surface marker cadherin E overlapped well in immunofluorescence (IF) stain with lung cancer cells and IHC stain in lung cancer patient tissue (Figure S1) and flow cytometry result suggests that SFPQ is on the cell surface (Figure S1C). This shows SFPQ presence on the cancer cell surface.

FIGURE 1Splicing factor proline and glutamine rich (SFPQ) is present not only in the nucleus of cancer cells. Immunohistochemistry (IHC) stain was performed on human cancer tissues. IHC was performed using antibodies to SFPQ to display SFPQ distribution in the normal adjacent region (left panel) and cancer region (middle panel); the enlarged images were presented at the right panels. The counterstain was Mayer's haematoxylin to display the nucleus (blue). Scale bar: 100 µm. The representative images from lung cancer (A), prostate cancer (C), kidney cancer (E), liver cancer (G) and breast cancer (I), SFPQ stain was quantified with ImageJ and summarised in (B), (D), (F), (H) and (J) sequentially. Twelve each of cancer patient specimens, five sections were imaged in each specimen. Lung cell PCS‐201‐020, kidney cell HEK293, prostate cell RWPE‐1 and bronchial cell HEBC3‐KT cells and liver cancer cell MHCC97, HepG2, kidney cancer cell Caki‐1, breast cancer cell MCF, MDA468, PC3 and prostate cancer cell DU145 were used to isolate subcellular fraction. (K) SFPQ level in cancer membrane fractions were analysed in Western blot (left panel). Densitometry values summarising Western blot data are shown in the right panel. Cadherin served as a loading control. (L) SFPQ level in cancer cytoplasm fractions were analysed in Western blot (left panel). Densitometry values summarising Western blot data are shown in the right panel. GAPDH served as a loading control. (M) SFPQ level in cancer nuclear fractions were analysed in Western blot (left panel). Densitometry values summarising Western blot data are shown in the right panel. Lamin served as a loading control. (N) Immunofluorescence (IF) stain was performed on lung cell PCS‐201‐020, kidney cell HEK293, prostate cell RWPE‐1 and bronchial cell HEBC3‐KT cells and liver cancer cell MHCC97, kidney cancer cell Caki‐1, lung cancer cell A549 and prostate cancer cell DU145 with SFPQ antibody 67129‐1‐Ig, Protech. Scale bar: 100 µM. (O) Anti‐SFPQ internalisation. Anti‐SFPQ 1A6 and normal mouse immunoglobulin G (IgG) were labelled with pHrodo Green (P36015, ThermoFisher) by following the manufacturer's instruction. The conjugates were then incubated with lung cancer H661 cells and the images were taken at indicated time points. Fluorescence iFL pHrodo is pH sensitive. Receptor‒ligand (SFPQ/antibody) internalised in the lysosome (lower pH) and the fluorescent colour gets stronger.

**Figure:**
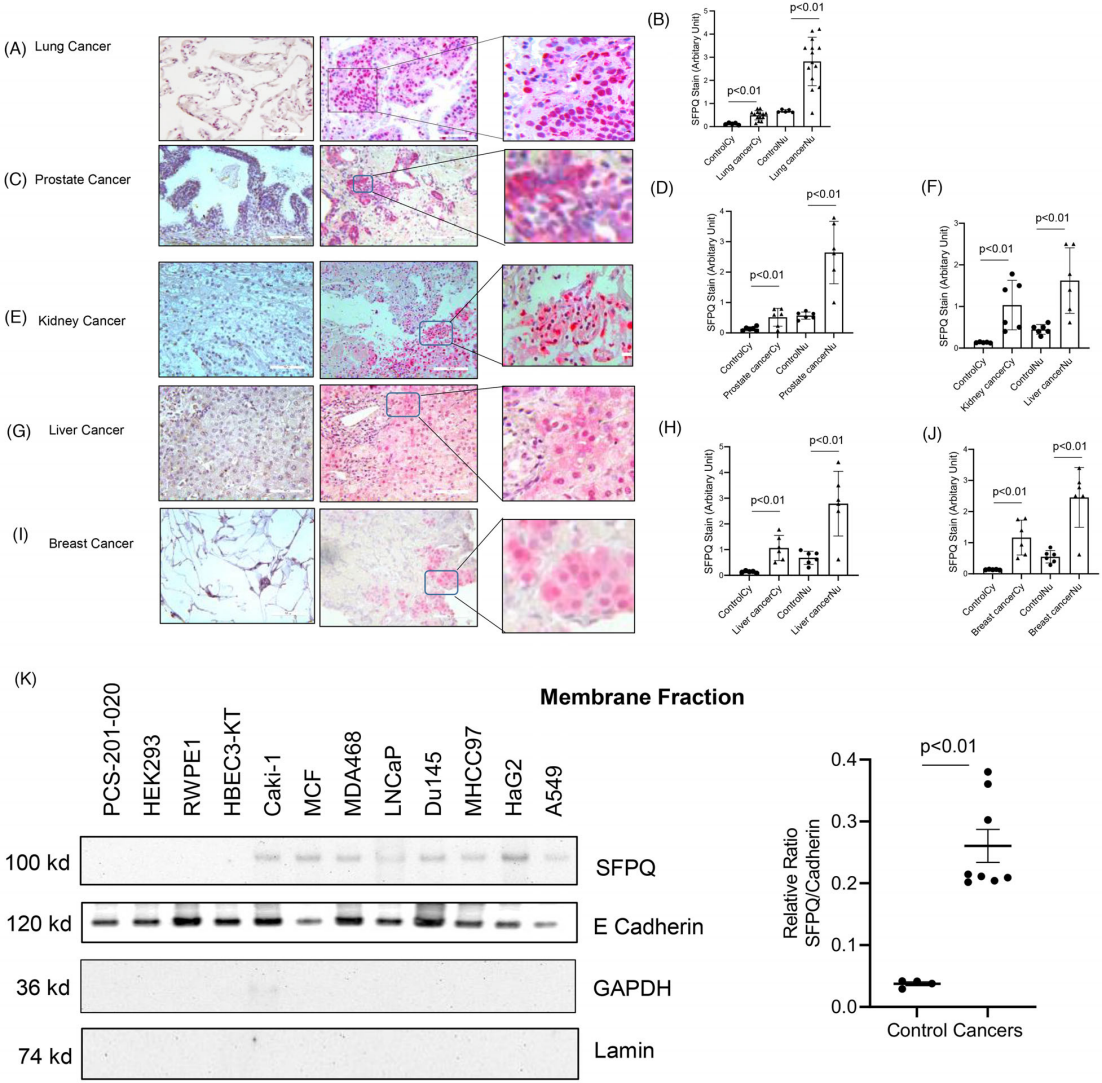

**Figure:**
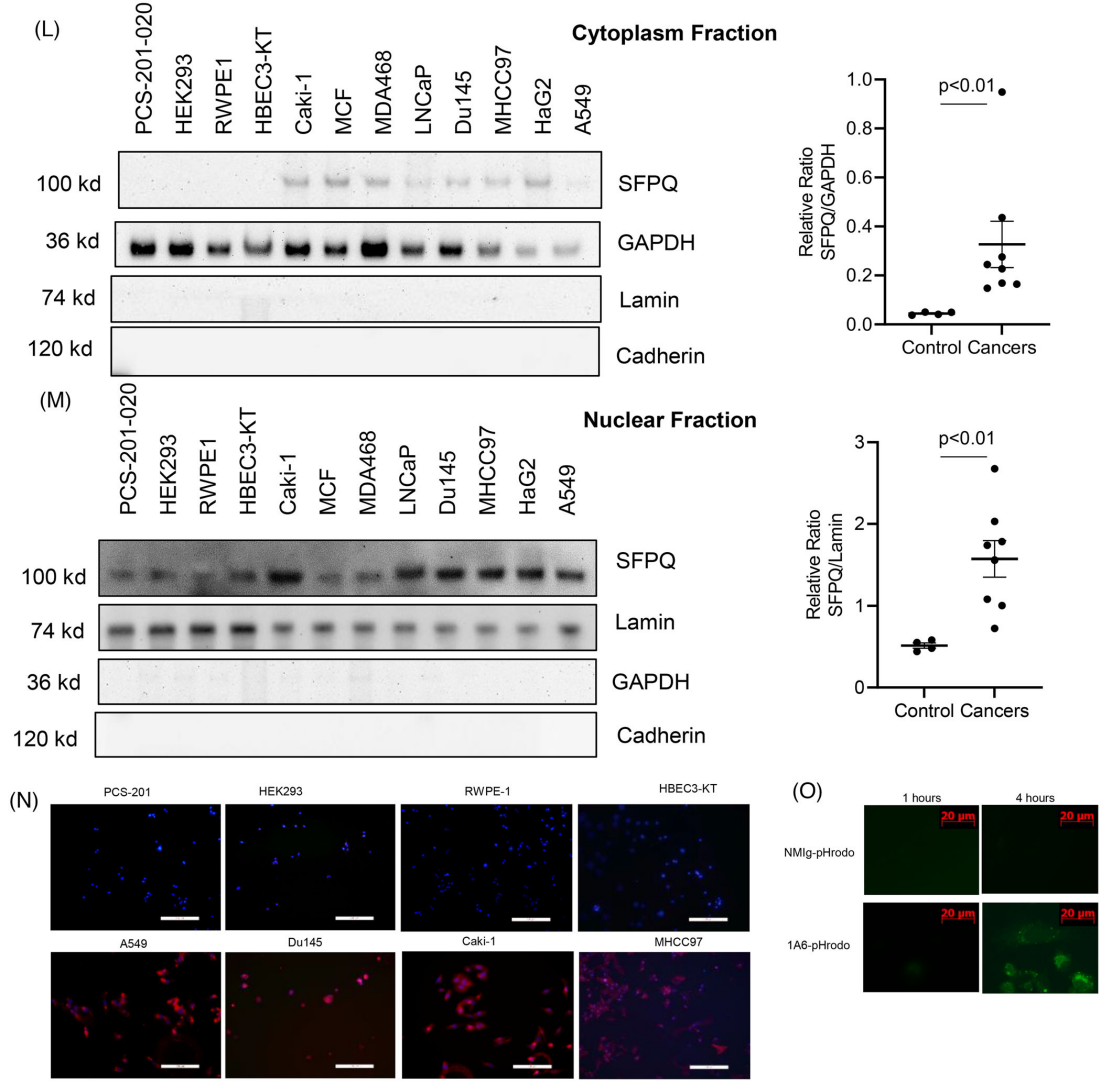

We then examine if interacting cell surface SFPQ affects cancer cell function. High SFPQ expressing lung cancer cells H661 (Figure 2A) were used to identify cell surface SFPQ binding antibodies, and 9/19 antibodies tested positive in IF stain (Figure 2B shows examples of them). Binding SFPQ could inhibit cell proliferation in lung cancer lines (Figure 2C) and other cancer cell lines from other cell types (Figure 2D). The cell proliferation marker ki67 expression levels are low in those proliferation‐inhibited cells (Figure 2E). The inhibition levels are consistent with the SFPQ membrane level (Figure 2A,C,D). Some antibodies also altered apoptosis (Figure 2F) and inhibited cell invasion (Figure 2G,H). When we applied some of those antibodies to other cancer cell lines, SFPQ antibody 1A6 inhibited cell proliferation (Figure S2) and invasion in those cancer cells (Figure S3) while knockdown the SFPQ in those cells reduced the antibody inhibition. These data indicate that different SFPQ antibodies affect cancer cells differently. In vivo study, lung cancer cells A549, H648 and H661 were intravenous; (IV) injected into NSG mice, and anti‐SFPQ 1A6 or control IgG was applied to the mice six times in intervals of 3 days. There are more cancer cell clusters in the normal, IgG‐treated mouse than the SFPQ antibody‐treated mouse in haematoxylin and eosin stain, SFPQ IHC analysis and human cell quantification (Figure 2I‒L). Together, these data indicate that SFPQ antibody treatment attenuates the ability of lung cancer cells to grow in vivo. These findings suggest that cell surface SFPQ could be a therapeutic target.

**FIGURE 2:**
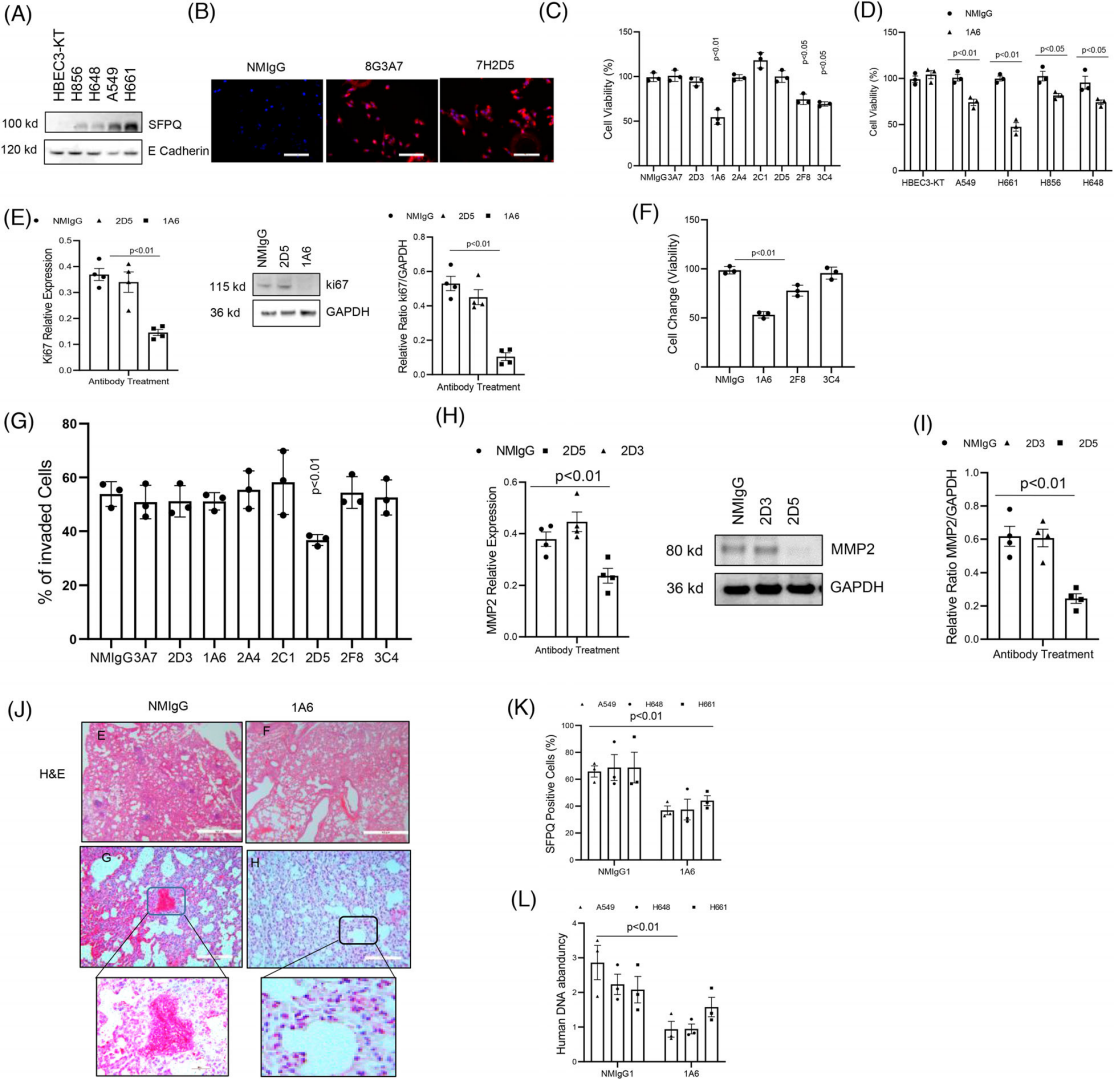
Antibody binding of cell surface splicing factor proline and glutamine rich (SFPQ) affects cancer cell function. Lung cancer cells were used to observe if antibody binding affects cell functions. (A) Lung cancer cell membrane fraction was used to analyse SFPQ level in Western blot analyses using lung NSCLC primary cell lines H661, H648 and H856 and ATCC bronchial cell HBEC3‐KT, lung cancer cell line A549. Antibody: SFPQ, 67129‐1‐Ig, Protech. (B) Immunofluorescence (IF) stain was performed on lung cancer cell H661 with normal mouse IgG (NMIgG), SFPQ antibody 8G3A7 and 7h2D5. Scale bar = 100 µM. (C) Lung cancer cells viability was assessed with anti‐SFPQ antibody treatment. H661, 10 µg/mL, 24 h. (D) Lung cancer cells A549, H661, H856 and H648 and bronchial cell HBEC3‐KT viability was assessed with anti‐SFPQ antibody 1A6., 24 h. (E) Cell proliferation marker ki67 expression was analysed with reverse transcription polymerase chain reaction (RT‐PCR) and Western blot. (F) Apoptosis assay was conducted in H661 lung cancer cell with SFPQ antibodies treatments. (G) Cell invasion assay was conducted in H661 lung cancer cell with SFPQ antibodies treatments. (H) Cell motion marker MMP2 was quantified in RT‐PCR and Western blot analyses using cell lines H661. Data are shown as mean ± SE. Three technical replicates were performed for all experiments. Lung cancer cells were treated with normal mouse IgG or SFPQ antibody 1A6. NOD scid gamma (NSG) mice implanted with lung cancer cells (A549, H856 and H661) via IV (3 × 10^5^ cells/50 µL); three mice per cell group. (I) Serial 4 µm sections of right lung tissue from lung cancer tumors implanted mice treated with normal mouse IgG (NMIgG, left panel) and SFPQ antibody 1A6 (1A6, right panel). Representative haematoxylin and eosin (H&E) (scale bar: 500 µm) and (J) IHC staining with SFPQ antibody 1A6 assessing GPR81 positivity. IHC for SFPQ was conducted to assess the distribution of human cells expressing SFPQ protein expressing cells from mice lungs from human lung tumours of A549, H846 and H661. Lower panel shows an enlarged image from the indicated area. Scale bar: 100 µM. (K) SFPQ‐positive cells in sections were quantified with Image J. (L) Cell quantification was conducted with quantitative polymerase chain reaction. (Q‐PCR). SFPQ binding affects lung cancer progression in vivo. NSG mice implanted with lung cancer cells (A549, H856 and H661) treated with either normal mouse IgG, or SFPQ antibody via IV (3 × 10^5^ cells/50 µL; 100 µg antibody); nine mice per group.

We performed an immunoprecipitation proteomics analysis of SFPQ with the cell membrane fraction to identify SFPQ interacting proteins. Proteomic analysis revealed many proteins bound to SFPQ (Table S1). By analysing their possible functions and signal transduction pathways via Ingenuity pathway analysis (https://digitalinsights.qiagen.com/products/qiagen‐ipa), these proteins were found to potentially include pathways of signal transduction that impact cell proliferation, apoptosis, metabolism, senescence and migration (Figure 3A,B and Tables S2 and S3). To confirm that these proteins interact with SFPQ, SFPQ antibody immunoprecipitation and Western blotting were performed with antibodies against PPIA, H2AX, S100A4 and VIM in the lung cancer cell membrane fraction (Figure 3C). PPIA, H2AX, S100A4 and VIM bands were present in the SFPQ pull‐down material, demonstrating that SFPQ binds to PPIA, H2AX, S100A4 and VIM in the cell membrane. IF double stain also shows SFPQ and S100A4 colocalised in cell membrane (Figure S5). The involvement of S100A4 in cancer cell migration has been reported previously^7^, ^8^, ^9^; we then examined the effect of the expression of SFPQ on S100A4 expression and cell invasion in solid cancer cells. SFPQ and S100A4 are higher in lung cancer cell lines (Figure 3D). SFPQ or S100A4 was knocked down with shRNA in lung cancer cells (with scrambled shRNA used as a control), and SFPQ and S100A4 expression levels and SFPQ/S100A4 complex were inhibited (Figure 3E,F). The cell invasion and migration marker MMP2 was reduced in those lung cancer cells with SFPQ or S100A4 knockdown (Figure 3G,H). We also observed if the SFPQ/S100A4 complex exists in other solid cancer cell lines. We found that SFPQ/S100A4 formed a complex in those cancer cells but not in normal bronchial cells (Figure S4). Knockdown SFPQ or S100A4 inhibited cell invasion in breast cancer cells (Figure S5). These indicate that membrane SFPQ/S100A4 affects cell invasion in cancer cells.

**FIGURE 3:**
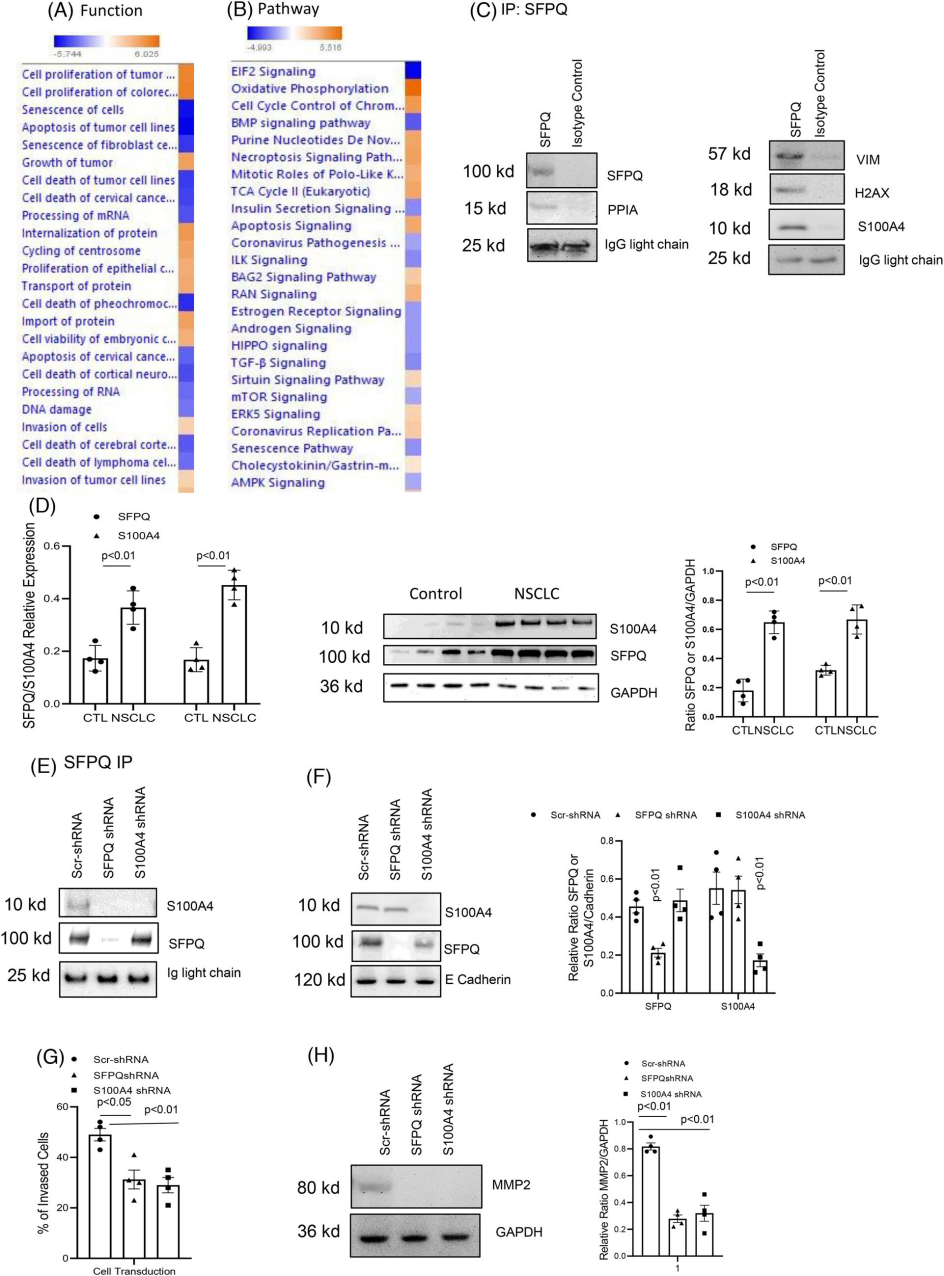
Interaction of splicing factor proline and glutamine rich (SFPQ) with membrane proteins affects lung cancer cell function. Proteins identified from GPR81 immunoprecipitated proteins in lung cancer cells in Proteomics analysis were applied to ingenuity pathway analysis (IPA). (A) Top functions associated with the lung cancer cell dataset as shown by IPA pathway analysis. (B) Top canonical pathways associated with proteins from lung cancer cell dataset as shown by IPA pathway analysis. Cell functions or pathways identified are represented on the *y*‐axis. The *x*‐axis corresponds to the –log of the *p*‐value (Fisher's exact test) and the orange points on each pathway bar represent the ratio of the number of proteins in a given pathway that meet the cutoff criteria, divided by the total number of proteins that map to that pathway. (C) Immunoprecipitation and Western blot analysis with GPR81 bound proteins. The nuclear fraction from A549 was used in immunoprecipitation with SFPQ antibody. Then, SFPQ, PPIA, S100A4 and VIM antibody were used in Western blot analysis with SFPQ pull‐down portion of lung cancer membrane fraction. The membrane SFPQ/S100A4 complex affects lung cancer cell invasion. (D) S100A4 and SFPQ RT‐PCRs were conducted with lung cancer cell H661, A549, H846 and H648 (left panel). GPR81 antibody was used in immunoprecipitation with those lung cancer cells and the SFPQ levels were measured with Western blot (middle panel). Densitometry values summarising Western blot data are shown in the right panel. GAPDH served as a loading control. Lung cancer cells transduced with Lenti virus with Scramble shRNA, SFPQ shRNA or S100A4 shRNA and the cell membrane fraction were used in (D‒H). (E) SFPQ immunoprecipitation was conducted with the cell membrane fraction from lung cancer cells. Then, the protein bands were detected with anti‐S100A4 and SFPQ antibodies. (F) Western blot analysis was conducted with cell membrane fraction from lung cancer cells. Then, the protein bands were detected with anti‐S100A4 and SFPQ antibodies. Western blot was shown in the left panel. Densitometry values summarising Western blot data are shown in the right panel. Cadherin served as a loading control. (G) Invasion assay was conducted with lung cancer cells. Lung cancer cells were transduced with Lenti virus with scramble shRNA, SFPQ shRNA or S100A4 shRNA. (H) The same set of cells were used to analyse MMP2 expression. Western blot (left panel). Densitometry values summarising Western blot data are shown in the right panel. GAPDH served as a loading control. Lung cancer cells A549, H661 and H856 were used in (D‒H).

Taken together, SFPQ is present on the cell surface and affects cell function via interaction with S100A4, PPIA, etc. in lung cancer and other solid cancer cells. Anti‐SFPQ antibodies could inhibit cancer cell proliferation and invasion by binding to SFPQ cells. Therefore, cell surface SFPQ may serve as a prognostic marker and therapeutic target for those cancers.

## AUTHOR CONTRIBUTIONS

Libang Yang and Robert Kratzke conceived, designed and directed the studies with input from Craig A. Henke, Sabine Karam and Scott M. Dehm. Libang Yang and Robert Kratzke wrote the manuscript with assistance from all the authors. Libang Yang, Adam Gilbertsen, Bo Sun, Yingming Li and Blake Jacobson established primary human cell lines, performed Q‐PCR, Western blot analysis, performed gain and loss of function experiments, mouse studies and immunohistochemistry.

## CONFLICT OF INTEREST STATEMENT

The authors declare they have no conflicts of interest.

## Supporting information



Supporting Information

## Data Availability

This manuscript represents original work, and all sources have been properly cited. Ethical approval was obtained and informed consent was secured where necessary. The data that support the findings of this study are available upon request from the corresponding author.
