# Supplementary material for: SFPQ subcellular mislocalisation entails altered cancer cell function
Source: Clin Transl Med. 2025 Aug 6;15(8):e70431. doi: 10.1002/ctm2.70431 (PMC12328246; doi:10.1002/ctm2.70431)
Supplement: Supplementary file 1 — Supporting Information [file CTM2-15-e70431-s001.docx]

**SFPQ Subcellular Mislocalization Entails Altered Cancer Cell Function**

Libang Yang^1^*, Adam Gilbertsen^1^, Blake Jacobson^2^, Robert Kratzke^2^, Yingming Li^3^, Bo Sun^4^, Sabine Karam^6^, Scott M. Dehm^3^, Craig A. Henke^1^

**Affiliations:**

^1^Department of Medicine, University of Minnesota, 420 Delaware Street, SE, Minneapolis, Minnesota 55455, USA

^2^Hematology, Oncology and Transplantation, School of Medicine, University of Minnesota, 420 Delaware Street, SE, Minneapolis, Minnesota 55455, USA

^3^Masonic Cancer Center, Department of Medicine, University of Minnesota, 420 Delaware Street, SE, Minneapolis, Minnesota 55455, USA

^4^Gastro, Hepatology, Nutrition Division, Department of Medicine, University of Minnesota, 420 Delaware Street, SE, Minneapolis, Minnesota 55455, USA

^5^Division of Nephrology. Department of Medicine, University of Minnesota, 420 Delaware Street, SE, Minneapolis, Minnesota 55455, USA

**Supplementary material**

**1. Supplementary** **methods and material**

**2. Supplementary discussion**

**3. Supplementary references**

**4. Supplementary figures**

**5. Supplementary tables**

**Supplementary Methods and Materials**

**Cancer cell lines, primary cell lines and patient tissue sections**.

Cell lines from the ATCC were used in this study. RWPE-1 and HEBC3-KT cells were cultured with Keratinocyte-SFM (Gibco 10724-011) and 0.1% soybean trypsin inhibitor according to the instructions provided by the ATCC. Lung cell PCS-201, prostate cell RWPE1, bronchial cell HBEC3-KT, kidney cell HEK-293, liver cancer cell MHCC97, HepG2, kidney cancer cell Caki-1, breast cancer cell MCF, MDA468, prostate cancer cell LNCaP3, and DU145 cells were cultured in DMEM supplemented with 5% FBS. Primary lung cell lines were established from patients who fulfilled the diagnostic criteria for lung cancer and other lung diseases, including a pathological diagnosis of usual interstitial pneumonia. Patient controls were selected to be similar in age to lung cancer patients with non-cancer lung disorders. Control and lung cancer cell lines were derived from lungs and cultivated as previously described (1). Human patient tissue sections were collected and prepared through Bionet, University of Minnesota. We utilized 4 non-cancer primary control cell lines from lung tissue not involved in the primary process: histologically normal lung tissue from a gunshot victim (*n* = 1) or chronic obstructive pulmonary disease (COPD) (*n* = 3) and 12 non-cancer patient tissue samples from patients with interstitial lung disease (n=5) or chronic obstructive pulmonary disease (COPD) (*n* = 7) for control tissue sections. A pathologist verified that all the tissue was tumor-free.

**Isolation of the cell cytoplasm, cell membrane, and nucleus** Primary cells and cancer cells were used. Subcellular fractions of cancer cells and control cells were isolated with subcellular fraction extraction reagents, Mem-PER Plus Membrane Protein Extraction Kit (89842) for membrane and Subcellular Protein Fractionation Kit (89840) for other fractions (Thermo Scientific, USA), following the manufacturer’s instructions.

**Monoclonal antibodies specific for SFPQ**

Mouse immunization and hybridoma generation were conducted by following previously published methods (2, 3). The antigen was purified native human SFPQ (4). It was verified with proteomics analysis and cross-verified with other commercial antibodies (EPR11846, Abcam; 67129-1-AP, Proteintech; PA5-29948, Thermo Fisher; NB100-61045, Novus Biologicals). It is also used to screen SFPQ antibody -producing hybridoma. Hybridomas that were found to be positive upon [ELISA](https://www.sciencedirect.com/topics/immunology-and-microbiology/enzyme-linked-immunosorbent-assay) screening on SFPQ-coated plates were further tested by [Western blotting](https://www.sciencedirect.com/topics/immunology-and-microbiology/western-blot) on recombinant [SFPQ](https://www.sciencedirect.com/topics/biochemistry-genetics-and-molecular-biology/nuclear-protein) (Abnova, Taiwan) and purified SFPQ (using SFPQ affinity column) from human cell lysate. Then, the [isotype](https://www.sciencedirect.com/topics/immunology-and-microbiology/isotype) of the eight positive clones was determined using the mAb-based [mouse](https://www.sciencedirect.com/topics/veterinary-science-and-veterinary-medicine/rat) [Ig](https://www.sciencedirect.com/topics/immunology-and-microbiology/intravenous-immunoglobulin) isotyping kit (BD Pharmingen). All antibodies were identified with Western blot and Immunofluorescence stain after a regular ELISA screen. Supplemental Figure 6 shows an example of one antibody identification. For SFPQ antibody confirmation, we further observe if it could detect the same protein as commercial antibodies. For instance, anti-SFPQ 2G1A6 binds SFPQ in cell lysates from HBEC3-KT and H661 and the purified human SFPQ in Western blot (Figure S6).

**SFPQ antibody purification**

SFPQ antibody was purified with a Protein G affinity column. Briefly, the collected medium was centrifuged (4000*g* for 5 min at 4 °C), and filtered for clarification (through a 0.45 μm filter), and the protein was purified by [affinity chromatography](https://www.sciencedirect.com/topics/medicine-and-dentistry/affinity-chromatography) using a [Sepharose](https://www.sciencedirect.com/topics/medicine-and-dentistry/sepharose) protein G (GE Healthcare) equilibrated in 20 mM Na-phosphate (pH 7.4,) and 500 mM NaCl. SFPQ antibody was eluted with 0.1 M glycine (pH2.8) and then neutralized with Tris-HCl (1M, pH8.5).  SFPQ antibodies were determined by Western blotting using SFPQ, pooled, concentrated, and buffer-exchanged into 20 mM Hepes (pH 7.2) and 150 mM NaCl using a Millipore Ultrafree-15 spin concentrator (4). The protein concentration was monitored by spectrometry (280 nm absorbance).

**Strong cation exchange (SCX) chromatography, LC-MALDI and 4800 MS/MS, and peptide and protein identification** Peptide/protein isolation and identification were conducted as described previously (5). Protein concentrations were determined in desalted samples with Bradford reagent (Bio-Rad, Hercules, CA), LC‒MS data was acquired for each concatenated fraction using an Easy-nLC 1000 HPLC (Thermo Scientific Inc., Waltham, MA) in tandem with a Thermo Fisher Orbitrap Fusion (Thermo Scientific Inc., Waltham, MA). Peptides were loaded directly onto a 75 cm × 100 µm internal diameter fused silica PicoTip Emitter (New Objective, Woburn, MA) packed in-house with ReproSil-Pur C18-AQ (1.9 µm particle, 120 Å pore; Dr. Maish GmbH Ammerbuch, Germany). The column was heated to 55 °C, and a flow rate of 300 µL/minute was applied during the gradient. The gradient was as follows: 5-22% Buffer B (A: 0.1% formic acid in water, B: 0.1% formic acid in acetonitrile) for 45 minutes, 22-35% B for 25 minutes, and 35-95% B for 10 minutes. The column was mounted in a nanospray source directly in line with an Orbitrap Fusion mass spectrometer (Thermo Scientific). The spray voltage was 2.1 kV in positive mode, and the heated capillary was maintained at 275 °C. The orbital trap was set to acquire survey mass spectra (380–1580 m/z) with a resolution of 60,000 at 100 m/z with automatic gain control (AGC) 1.0E6 and 250-ms min injection. EASY-IC was selected for internal mass calibration. The 12 most intense ions (2-7 charged states) from the full scan were selected for fragmentation by higher-energy collisional dissociation with a normalized collision energy of 35% and detector settings of 60k resolution, AGC 5E4 ions, a 250 ms maximum injection time, and an FT first mass mode fixed at 110 m/z. Dynamic exclusion was set to 40 s with a 10 ppm high and low mass tolerance.

**Database searching for protein identification** The tandem mass spectra were analyzed using Sequest (XCorr Only) in Proteome Discoverer 2.4.0.305 (Thermo Fisher Scientific, Waltham, MA). We used the UniProt human universal proteome (UP000005640) sequence database from July 12, 2022, merged with the common laboratory contaminant protein database from <https://www.thegpm.org/crap/>, for database searching. The sequence search parameters included the following: trypsin enzyme, fragment ion mass tolerance of 0.1 Da, precursor ion tolerance of 20 ppm, carbamidomethyl cysteine as a fixed modification, pyroglutamic acid from glutamine, deamidation of asparagine, oxidation of methionine and N-terminal protein acetylation as variable modifications.

**Ingenuity Pathway Analysis (IPA)** The lung cancer MSC nuclear proteomic analysis data were imported to IPA (http://[www.ingenuity.com](http://www.ingenuity.com), 2024, Nov) for functional analysis, canonical pathway analysis, and upstream regulator analysis. Fisher’s exact test was used to calculate a *P* value, which determines the probability that each biological function and/or disease assigned to the dataset is caused only by chance (6).

**Cell viability assay** Cell viability was measured using CellTitle-blue kits (Promega, USA). Single-cell suspensions of 2×10^4^ cells were cultured in 96-well plates with blue reagent for 16 hours, and the cells were quantified according to the manufacturer’s instructions. The results were quantified with a SpectraMax M3 microplate reader (Molecular Devices).

**The cell invasion assay** A cell invasion assay was conducted with Transwell inserts (8 µm pore) in 24-well tissue culture plates (Millipore, USA). Cancer and control cells were cultivated in serum-free DMEM for 24 h, trypsinized, and inoculated into the upper chamber at 2 X10^4^ cells/well in 300 µl of serum-free DMEM. The lower chamber contained 500 µl of 10% FBS DMEM (positive control), conditioned DMEM, or serum-free DMEM (negative control). After 16 h at 37°C, the MSCs were detected with CyQuant GR Dye. The cells remaining in the upper chamber were removed with a release buffer, and the cells that migrated across the insert were quantified with a fluorescence reader.

**Apoptosis assay** A cell apoptosis assay was conducted with CellEvent Caspase3/7 kit (Thermo Fisher, USA). Briefly, cancer and control cells were cultivated in 10% FBS DMEM with treatment. After 24 h at 37°C, the cells were washed and incubated with Caspase dye for 30 minutes, then results were quantified with a fluorescence reader.

## **Antibody internalization assay** pHrodo dye is pH sensitive and could be used to specifically detect endocytosis and phagocytosis. pHrodo Green (P36015, Thermo Scientific, USA) was used to demonstrate SFPQ internalization. SFPQ antibody was labeled with pHrodo by following manufacturer’s instructions. The conjugates then were incubated with NSCLC cancer cells, which has SFPQ expressing on the cell surface, antibody binds to SFPQ and is transported into the cytosol by endocytosis, where the lower pH sensitizes the fluorescence. The green color in the cell indicates the internalized antibody.

**Plasmids/Constructs**. For the loss-of-function assay, SFPQ was knocked down using shRNA (pGIPZ-SFPQ shRNA; IDT and UMN Genomics Center). S100A4 was knocked down using shRNA (pGIPZ-S100A4 shRNA; Applied Biological Materials Inc., Canada). Scrambled shRNA served as the control. Cells were transduced with a lentiviral vector containing shRNAs and polybrene (7).

**Western blot analysis** Cells were washed twice in cold PBS and lysed in New RIPA lysis buffer (150 mM NaCl, 50 mM Tris pH 8.0, 1 mM EDTA, 1 mM EGTA, 0.5 % sodium deoxycholate, 0.1 % SDS, and 1 % Triton X-100) (Immunoprecipitation for Proteomics the RIPA only with 0.1% SDS not Triton X-100). supplemented with protease inhibitor cocktail (0.1 M phenylmethylsulfonyl fluoride, 5 μg/ml leupeptin, 2 μg/ml aprotinin, and 1 μg/ml pepstatin). The protein concentrations of the whole-cell lysates were determined using the BCA method, and equal amounts of each protein sample (15 μg) were separated on an 8~14 % SDS–polyacrylamide gel at 80 V. The separated proteins were then transferred to a polyvinylidene difluoride membrane for 8 minutes on a Turbo transfer system (Invitrogen, USA). After blocking with 5 % skim milk powder for 1 h at RT, the membrane was incubated with primary antibody for 1 h at RT or overnight at 4 °C. The membrane was washed three times for 15 min with 0.05 % PBS-Tween and then incubated for 1 h at RT with a horseradish peroxidase-conjugated secondary antibody. After extensive washing with 0.05 % PBS-T, protein bands were visualized by ECL Plus according to the manufacturer’s instructions (Cell Signaling Technology, USA).

**Real-time reverse transcription PCR** Total RNA was extracted with the RNeasy Mini Kit, and cDNA was synthesized with the miScript92 RT Kit (Qiagen). The PCRs contained 10 μl of SYBR Green SuperMix (Bio-Rad), 900 nM forward primer, 900 nM reverse primer, and 50 ng of cDNA in a 20 μl reaction volume. GAPDH was used as a reference, and the expression of GAPDH was normalized to 1. Reactions were performed in an A7900 HT Sequence Detector (Applied Biosystems) with a cycling protocol described previously (Applied Biosystems) (8). The primers used were as follows:

GAPDH Forward: 5′- TGTTGCCATCAATGACCCCTT-3′

GAPDH Reverse: 5′-CTCCACGACGTACTCAGCG-3′

S100A4 Forward: 5′-GTACGTGTTGATCCTGACTGCTGTCATGG-3′

S100A4 Reverse: 5′-TCATTTCTTCCTGGGCTGCTTATCTGGG-3′

Ki67 Forward: 5′-TCCTTTGGTGGGCACCTAAGACCTG-3’

Ki67 reverse: 5′- TGATGGTTGAGGTCGTTCCTTGATG-3’;

MMP2 Forward: 5′-CTCAGATCCGTGGTGAGATCT-3′

MMP2 reverse: 5′-CTTTGGTTCTCCAGCTTCAGG-3′

SFPQ Forward: 5’-GATCTACAGGGAAAGGCATTGTTG-3’

SFPQ Reverse: 5’-GATACATTGGATTCTTCTGGGCA-3’

RT–PCR products were quantified at the log-linear portion of the curve using LightCycler analysis software and compared to an external calibration standard curve.

**Mouse xenograft model of cancer**.

We utilized NOD/SCID/IL2rγ/B2M (NSG) mouse model to assess the metastatic ability of NSC lung cancer cells *in vivo* (9). Mice were housed under pathogen-free conditions in the University of Minnesota Molecular and Cellular Center Isolation Facility. All mouse studies followed the protocols reviewed and approved by the University of Minnesota Institutional Animal Care and Use Committee (IACUC). An average of 10 weeks of age-matched NSG male and female mice (Jackson Laboratories) were used for intravenous injections for cancer studies. 3 days before IV cell injection, the mice were irradiated (225 cGy) to deplete immune cells. 5X10^5^ of lung cancer cells suspended in 50 μL PBS were IV injected into the mice tail with a 28-gauge needle after mice were anesthetized with 5% isoflurane. For treatment with antibodies, 100 ug of SFPQ antibody (1A6) or control mouse IgG (Bio X Cell) was injected intraperitoneally on day 4, 7, 10, 13, 16, 19 after cancer cell inoculation. All experimental mice were monitored until fully recovered from anesthesia, and were subsequently monitored for disease progression by measuring body weight and behavior signs (pain and distress, et al) daily. Mice were euthanized by CO_2_ and different organ tissues were harvested 4 weeks later. Histological (H&E and trichrome staining) and immunohistochemical analysis was performed on paraffin embedded mouse tissues.

**Quantification of human lung cancer cells in mouse lung tissue**. Mice were euthanized 4 weeks after the adoptive transfer of human cells, and the lungs were harvested. The lungs were digested, and genomic DNA was isolated using a PureLink Genomic DNA Mini Kit according to the manufacturer’s instructions (Invitrogen). Real-time PCR was used to quantify human cancer cells in the mouse lungs by measuring the amount of human-specific DNA sequence using human specific primers per a previously published protocol (10). The PCR assay was performed for 40 cycles using the human genomic DNA–specific primers (forward: 5’-ATGCTGATGTCTGGGTAGGGTG-3’; reverse: 5’-TGAGTCAGGAGCCAGCGTATG-3’). Genomic DNA from 1 X 10^6^ A549 was used as reference control in qPCR. For image quantification with Image J, for calculation, Area Mean StdDev Mode; Min Max IntDen, Median %Area RawIntDen. We followed the method described before(11).

**Statistical analysis** All experiments were performed at least in triplicate, and the results were analyzed using the Student’s t-test or two-way ANOVA (for proteomics methods described above). The criterion for significance was p<0.05. Numerical data are reported as the means ± standard deviations.

**Study approval**. De-identified patient samples were obtained by our tissue procurement service (Bionet) under a waiver of informed consent from the University of Minnesota Institutional Review Board (University of Minnesota IRB ID: 1504M68341). Animal protocols were approved and conducted in accordance with the University of Minnesota Institutional Animal Care and Use Committee regulations (approval #1706-34890A).

**Supplementary Discussion**

Protein mislocalization is an important characteristic of many proteins in a range of human malignancies, which is defined as alterations in the proper subcellular localization of proteins (12). Protein mislocalization has important implications for alterations in the activation conditions, biological functions, and interaction networks of proteins. In particular, the aberrant localization of tumor suppressor proteins and proto-oncoproteins can alter their functions by either suppressing or supporting cancer initiation in normal cells, increasing cancer development, metastasis, and drug resistance (12, 13). In clinical diagnostic and prognostic applications, the capacity of malignant cells to have distinct subcellular protein localizations may be an intelligent tactic for identifying malignant cells in contrast to normal cells. For instance, fibromodulin, a proteoglycan that resides in the extracellular matrix, is irregularly located on the cell surface of chronic lymphocytic leukemia (CLL) cells but not in normal samples; thus, a new CLL diagnostic biomarker appropriate for cell surface flow cytometric detection might be suggested (14, 15). Moreover, direct targeting of the locations in which proteins accumulate in cancer cells and selection of the structural dissimilarities of proteins by biological weapons help us to specifically eradicate malignant but not normal cells.

SFPQ interacts with many proteins and further affects cell function in cancer cells (16-18). SFPQ is a multifunctional protein. It can bind to DNA and RNA and thus regulate RNA splicing and protein transcription. It is highly expressed in many cancers and plays a critical role in those cancers (19-21). It was also discovered that its accumulation in the cytoplasm could be the cause of some neuronal diseases (18). SFPQ cancer studies are relatively few, and no subcellular SFPQ has been related to cancer development yet. SFPQ levels are elevated not only in lung cancer but also in other cancer samples. SFPQ levels were greater in lung cancer cells than in other cells. Because SFPQ has multiple functions in cells, changes in its expression level may have an impact on cell function. The same is true for its membrane and cytoplasmic distribution. First, we found that its cytoplasmic isoform is exclusively present in high-stage solid cancers (21). We discovered that SFPQ is present in the membrane and cytoplasm fractions of cancer cells but not in the control cells. Its levels in the membrane and cytoplasm vary between cancer cell lines. For example, in the cell membrane fraction, its level is high in lung cancer H661 cells but lower in lung cancer H856 cells. It is expressed at high levels in the prostate cell line Du145 and liver cancer cell line MHCC97 but at lower levels in the kidney cell line Caki-1. It presents at similar levels in the cytoplasm of these cancer cell lines. When IHC and cell fraction experiments only suggest SFPQ may be present in the cell membrane, our IF and protein internalization results demonstrate that SFPQ is present on the cell surface which makes it a potential therapy target. When nuclear SFPQ affects cell function in cancer cells, its presentation in the cell membrane and cytoplasm may affect cell function too, where it may interact with other proteins. Proteomic analysis is revealed that SFPQ interacts with a number of proteins related to cell functions, and these proteins potentially include pathways involved in signal transduction that impact cell apoptosis, metabolism, senescence, migration, and proliferation, according to Ingenuity signal transduction analysis. When we treat the cancer cells with SFPQ antibodies, some of the antibodies affect cell viability, and some of them inhibit cell invasion, suggesting interacting with different parts of membrane SFPQ might trigger different signal transduction pathways and lead to different cell function changes. For example, when anti-SFPQ 1A6 caused apoptosis in H661 while 2D5 did not.

The cell surface SFPQ could serve as a therapy candidate since it is a nuclear protein. To observe if interacting with SFPQ affects cancer cell functions, we studied SFPQ antibodies. Screened SFPQ antibodies binding cancer cell surface we found there are only half of them are positive with lung cancer cells H661 and A549, 9/19 (7 from commercial companies, data not shown). Some of those antibodies affected cancer cell functions (Figure 2), which suggests cell surface SFPQ could serve as therapy targets. Since those antibodies were identified by ELISA, western blot analysis, immunofluorescence stain, IHC and some of those were positive in internalization assay and effective in cell function assays as therapy agents (22) those antibodies are a potential therapy for cancers.

S100A4 is a protein widely overexpressed in many tumor cells, including cancer stem cells, and is a critical regulator of cell-matrix adhesion, cell growth, EMT, and tumor progression. S100A4 is frequently expressed primarily on stem cells and cancer cells and is thought to contribute to cancer development and progression (23-25). S100A4 is involved in cancer cell function via multiple-level regulation, but membrane S100A4 is considered to be involved in cancer metastasis (25-27). When SFPQ still affects cell function via its regulation of nuclear structure, it may change cancer cell function and signal transduction by interacting with other proteins in the cell membrane. We observed that SFPQ binds to S100A4 in the cell membrane. Knocking down SFPQ or S100A4 inhibited the SFPQ/S100A4 complex in the membrane in cancer cells and cell invasion. Those suggest cell membrane SFPQ is involved cell invasion. How the membrane SFPQ affects cell functions needs further investigation.

Taken together, this study uncovers that SFPQ appears on the cancer cell surface. We found that interacting with SFPQ affected cancer cell function, though further research is needed to clarify the underlying mechanisms. These results offer insight into SFPQ's therapeutic potential and provide a foundation for future clinical applications.

**Supplementary References**

1. Zhang DG, Jiang AG, Lu HY, Zhang LX, Gao XY. Isolation, cultivation and identification of human lung adenocarcinoma stem cells. Oncol Lett. 2015;9(1):47-54.

2. Holzlohner P, Hanack K. Generation of Murine Monoclonal Antibodies by Hybridoma Technology. J Vis Exp. 2017(119).

3. Hyung-Yong Kim AS, Mina J. Izadjoo. Immunization, Hybridoma Generation, and Selection for Monoclonal Antibody Production. Vincent Ossipow NF, editor: Springer Products; 2014. 33-45 p.

4. Yang L, Gilbertsen A, Jacobson B, Kratzke R, Henke CA. Serum Splicing Factor Proline- and Glutamine-Rich Is a Diagnostic Marker for Non-Small-Cell Lung Cancer and Other Solid Cancers. Int J Mol Sci. 2024;25(16).

5. Libang Yang1* AG, Karen Smith1, Hong Xia1, LeeAnn Higgins2, Candace Guerrero2, Craig A. Henke1. Proteomic Analysis of the IPF Mesenchymal Progenitor Cell Nuclear Proteome Identifies Abnormalities in Key Nodal Proteins That Underlie Their Fibrogenic Phenotype. Proteomics. 2022.

6. Yang J, Gourley GR, Gilbertsen A, Chen C, Wang L, Smith K, et al. High Glucose Levels Promote Switch to Synthetic Vascular Smooth Muscle Cells via Lactate/GPR81. Cells. 2024;13(3).

7. Yang L, Xia H, Smith K, Gilbertsen A, Beisang D, Kuo J, et al. A CD44/Brg1 nuclear complex confers mesenchymal progenitor cells with enhanced fibrogenicity in idiopathic pulmonary fibrosis. JCI Insight. 2021;6(9).

8. Yang L, Geng Z, Nickel T, Johnson C, Gao L, Dutton J, et al. Differentiation of Human Induced-Pluripotent Stem Cells into Smooth-Muscle Cells: Two Novel Protocols. PLoS One. 2016;11(1):e0147155.

9. Gu JJ, Hoj J, Rouse C, Pendergast AM. Mesenchymal stem cells promote metastasis through activation of an ABL-MMP9 signaling axis in lung cancer cells. PLoS One. 2020;15(10):e0241423.

10. Yang L, Gilbertsen A, Xia H, Benyumov A, Smith K, Herrera J, et al. Hypoxia enhances IPF mesenchymal progenitor cell fibrogenicity via the lactate/GPR81/HIF1alpha pathway. JCI Insight. 2023;8(4).

11. Stossi F, Singh PK. Basic Image Analysis and Manipulation in ImageJ/Fiji. Curr Protoc. 2023;3(7):e849.

12. Ghaemimanesh F. The Protein Subcellular Mislocalization in Human Cancers. Avicenna J Med Biotechnol. 2020;12(1):1.

13. Wang X, Li S. Protein mislocalization: mechanisms, functions and clinical applications in cancer. Biochim Biophys Acta. 2014;1846(1):13-25.

14. Heinemeier KM, Skovgaard D, Bayer ML, Qvortrup K, Kjaer A, Kjaer M, et al. Uphill running improves rat Achilles tendon tissue mechanical properties and alters gene expression without inducing pathological changes. J Appl Physiol (1985). 2012;113(5):827-36.

15. Mayr C, Bund D, Schlee M, Moosmann A, Kofler DM, Hallek M, et al. Fibromodulin as a novel tumor-associated antigen (TAA) in chronic lymphocytic leukemia (CLL), which allows expansion of specific CD8+ autologous T lymphocytes. Blood. 2005;105(4):1566-73.

16. Stagsted LVW, O'Leary ET, Ebbesen KK, Hansen TB. The RNA-binding protein SFPQ preserves long-intron splicing and regulates circRNA biogenesis in mammals. Elife. 2021;10.

17. Taylor R, Hamid F, Fielding T, Gordon PM, Maloney M, Makeyev EV, et al. Prematurely terminated intron-retaining mRNAs invade axons in SFPQ null-driven neurodegeneration and are a hallmark of ALS. Nat Commun. 2022;13(1):6994.

18. Widagdo J, Udagedara S, Bhembre N, Tan JZA, Neureiter L, Huang J, et al. Familial ALS-associated SFPQ variants promote the formation of SFPQ cytoplasmic aggregates in primary neurons. Open Biol. 2022;12(9):220187.

19. Klotz-Noack K, Klinger B, Rivera M, Bublitz N, Uhlitz F, Riemer P, et al. SFPQ Depletion Is Synthetically Lethal with BRAF(V600E) in Colorectal Cancer Cells. Cell Rep. 2020;32(12):108184.

20. Kok VJT, Tang JY, Eng GWL, Tan SY, Chin JTF, Quek CH, et al. SFPQ promotes RAS-mutant cancer cell growth by modulating 5'-UTR mediated translational control of CK1alpha. NAR Cancer. 2022;4(3):zcac027.

21. Libang Yang1 AG, Blake Jacobson2, Naomi Fujioku2, Jenny Pham3, Craig A. Henke1, Robert Kratzke2. SFPQ and its isoform as Potential Biomarker for Non Small Cell Lung Cancer. Int. J. Mol. Sci2023.

22. Laflamme C, McKeever PM, Kumar R, Schwartz J, Kolahdouzan M, Chen CX, et al. Implementation of an antibody characterization procedure and application to the major ALS/FTD disease gene C9ORF72. Elife. 2019;8.

23. Hemandas AK, Salto-Tellez M, Maricar SH, Leong AF, Leow CK. Metastasis-associated protein S100A4--a potential prognostic marker for colorectal cancer. J Surg Oncol. 2006;93(6):498-503.

24. Ismail TM, Zhang S, Fernig DG, Gross S, Martin-Fernandez ML, See V, et al. Self-association of calcium-binding protein S100A4 and metastasis. J Biol Chem. 2010;285(2):914-22.

25. Jaiswal JK, Nylandsted J. S100 and annexin proteins identify cell membrane damage as the Achilles heel of metastatic cancer cells. Cell Cycle. 2015;14(4):502-9.

26. Sun H, Wang C, Hu B, Gao X, Zou T, Luo Q, et al. Exosomal S100A4 derived from highly metastatic hepatocellular carcinoma cells promotes metastasis by activating STAT3. Signal Transduct Target Ther. 2021;6(1):187.

27. Mathisen B, Lindstad RI, Hansen J, El-Gewely SA, Maelandsmo GM, Hovig E, et al. S100A4 regulates membrane induced activation of matrix metalloproteinase-2 in osteosarcoma cells. Clin Exp Metastasis. 2003;20(8):701-11.

**Supplementary** Figures

**Supplemental Figure 1 SFPQ present on cell membrane with cadherin E in IF and IHC**. **A**. NSCLC cell H661 cultured on coverslips were stained with cadherin E (ab1416, Abcam, USA) and SFPQ antibody (2G1A6). Images were captured using Leica LAS v4.4 software. Scale bar=20 µM. Representative images are shown. **B**. Immunohistochemistry (IHC) stain was performed on human lung tissue. NSCLC tissue sections were used in IHC stain. IHC was performed using antibodies for cadherin E (ab1416, Abcam, USA) and SFPQ (2G1A6) to display cadherin E distribution in red and SFPQ in light brown in cancer tissue section; Scale bar = 20 µm. **C**. FACS was conducted with Anti-SFPQ in NSCLC cell H661 and lung cell HBEC-3KT. Anti-SFPQ-PE(2G1A6) and PE mouse IgG1 Isotype were used in the cell stain (4X10^5^ each group). A set of representive flow cytometry images show in the left panel. The data was normalized to Isotype PE control.

**Supplemental Figure 2. Antibody binding of cell surface SFPQ affects cancer cell viability. A.** Cancer and normal cells were used to observe if antibody binding affect cell viability. Cell viability was assessed when the cell were incubated with anti-SFPQ antibody. Kidney cell HEK293, kidney cancer cell Caki-1, prostate cancer cell Du145, liver cancer cell MHCC97 and breast cancer cell MDA231 were used in this cell viability assay, anti-SFPQ antibody 1A6. Normal mouse IgG as control, 10 µg/ml, 24 hours. **B**. H661 transduced with scramble and SFPQ shRNA lentivirus and used in cell viability assay with SFPQ antibody (1A6, normal mouse IgG as control, 10 µg/ml, 24 hours) treatment.

**Supplemental Figure 3. Antibody binding of cell surface SFPQ affects cancer cell invasion. A.** Cancer and normal cells were used to observe if antibody binding affect cell invasion. Cell invasion was assessed when the cell incubated with anti-SFPQ antibody. Kidney cell HEK293, kidney cancer cell Caki-1, prostate cancer cell Du145, liver cancer cell MHCC97 and breast cancer cell MDA231 were used in this cell invasion assay, anti-SFPQ antibody 2D5. Normal mouse IgG as control, 10 µg/ml, 24 hours. **B**. H661 transduced with scramble and SFPQ shRNA lentivirus and used in cell invasion assay with SFPQ antibody (1A6, normal mouse IgG as control, 10 µg/ml, 24 hours) treatment.

**Supplemental Figure 4**. **The interaction of membrane SFPQ with S100A4 in cancer cells.** Cell membrane fractions were isolated from cancer cell lines to observe SFPQ and S100A4 interaction with immunoprecipitation. Anti-SFPQ (PA5-29948, Thermo Scientific, USA) was used in Immunoprecipitation and Western blot analysis with SFPQ (67129-1-Ig, Proteintech, USA) and S100A4 (1482, Abcam, USA) antibodies.

**Supplemental Figure 5. The membrane SFPQ/S100A4 complex affects Cancer cell invasion.**  Breast cancer cell MDA231 were transduced with Lenti virus with Scramble shRNA, SFPQ shRNA or S100A4 shRNA and the cell membrane fraction were used in this figure**. A**. Anti-SFPQ (PA5-29948, Thermo Scientific, USA) was used in Immunoprecipitation and Western blot analysis was conducted with SFPQ (67129-1-Ig, Proteintech, USA) and S100A4 (124805, Abcam, USA) antibodies (Left panel). Right panel: Western blot analysis with the same set samples, Cadherin as loading marker. **B.** Invasion assay was conducted with Breast cancer cell MDA231. Invasion ability was reduced in cancer cells transduced with Lenti virus SFPQ shRNA or S100A4 shRNA comparing to scramble shRNA. **C**. The same set cells were used to analyze MMP2 expression in Western blot analysis. GAPDH served as a loading control. D. Immunofluorescent stain shows that SFPQ and S100A4 colocalized in some subcellular locations. Antibodies same as A.

**Supplemental Figure 6 SFPQ antibody 2G1A6 confirmation with Western blot and IF**. A. SFPQ antibody 1A6 was used in Western blot with a variety of human samples. B. NSCLC cell H661 cultured on coverslips were fixed with 4% PFA and stained with the indicated antibodies followed by FITC-conjugated secondary antibody. Images were captured using Leica LAS v4.4 software. Bar=100 µM. Representative images are shown.

**Supplementary Tables**

**Table 1 Membrane SFPQ Interacting Proteins**

| ADH1 | HS90A | PRDX1 | DESP |
| --- | --- | --- | --- |
| ADT2 | HSP7C | PROF1 | DSC1 |
| ANXA2 | IF5A1 | Q32Q12 | FILA |
| ARF3 | IGHG4 | RAN | G3P |
| ATPA | J3KND3 | RL13A | G6PD |
| BOYJC5 | K1C14 | RL6 | GRP75 |
| BAF | K1M1 | RS14 | HOYN42 |
| BIP | K22E | S10A4 | S10A6 |
| CASA1 | LDHA | S10A9 | S10A7 |
| CASB | LDHB | SBSN | S10A8 |
| COF1 | LEG1 | SMD3 | CD44 |
| COX41 | LEG7 | SPB3 | K2M4 |
| DCD | LMNA | SPR1B | K1C6 |
| DSG1 | LORI | SSBP | ADT3 |
| EF1A1 | MYH9 | TBA1B | ARF4 |
| F5H2Z3 | MYH10 | TBB4 | H2C1 |
| F8VZU9 | NDKA | TCP4 | RS25 |
| FLNA | NPM | TGM1 | RS8 |
| H2A2B | PHB2 | THIO | RL22 |
| H2AW | PIGR | TKT | RL13A |
| H2AX | PIP | TRY1 | KRT85 |
| H2AY | PLAK | TRYP | KPRP |
| H2AZ | PLEC | VIME | KPRM |
| HNRPK | POTEE | ZA2G |  |
| HS71B | PPIA | S10A5 |  |

Proteins Identified by Mass Spectrometry (SFPQ bound proteins: Immunoprecipitation with SFPQ antibody). Each ID was decided with two or more unique peptides at 99% confidence.

**Supplemental Table 2** Top 50 cell function SFPQ binding protein involved

| Categories | p-value | Activation z-score |
| --- | --- | --- |
| Cellular Assembly and Organization and Maintenance | 5.62E-14 | 2.509 |
| Cancer,cell growth | 5.70E-14 | 2.585 |
| Senescence of cells | 3.52E-11 | -3.368 |
| Apoptosis of cancer cells | 3.75E-11 | -4.361 |
| Senescence of fibroblast | 3.94E-11 | -3.273 |
| Growth of tumores | 4.92E-11 | 1.254 |
| Cell Death and Survival | 5.85E-11 | -2.889 |
| Cell Death of prostate cancer | 5.86E-11 | -1.692 |
| Porocessing of mRNA | 7.88E-11 | -1.152 |
| Internalization of protein | 9.88E-11 | 0.226 |
| Cycling of centrosome | 1.10E-10 | 0.431 |
| Proliferation of epithelial cell | 1.63E-10 | 0.437 |
| Transportation of protein | 1.83E-10 | 0.283 |
| Cell death of pheochromocytoma | 1.87E-10 | -1.655 |
| Import of protein | 2.16E-10 | 0.437 |
| Cell viability of embryonic cells | 2.24E-10 | 0.275 |
| Apoptosis of cervical cancer | 2.53E-10 | -0.288 |
| Cell Death of cortical neurodoma | 4.19E-10 | -0.788 |
| Processing of RNA | 4.42E-10 | -0.559 |
| DNA damage | 4.48E-10 | -0.379 |
| Invasion of cells | 6.22E-10 | 0.474 |
| Cell Death of cerebral cortex | 6.77E-10 | -0.707 |
| Cell Death of lymphoma cells | 9.23E-10 | -0.849 |
| Invasopn of tumor cell lines | 4.84E-09 | 1.335 |
| Cellular Assembly and Organization | 8.47E-09 | 1.187 |
| Protein Synthesis | 8.73E-09 | 0.437 |
| Cellular Assembly and Organization | 3.35E-08 | 0.053 |
| Cancer,Organismal Injury and Abnormalities | 3.61E-08 | 0.279 |
| Cell Death and Survival | 5.22E-08 | -0.152 |
| Cellular Movement | 5.29E-08 | 0.202 |
| Neurological Disease,Organismal Injury and Abnormalities | 6.44E-08 | -0.703 |
| Cellular Assembly and Organization | 6.73E-08 | -0.35 |
| Infectious Diseases | 9.45E-08 | -0.039 |
| Cancer,Organismal Injury and Abnormalities | 1.27E-07 | -0.152 |
| Hematological Disease,Organismal Injury and Abnormalities | 1.58E-07 | -0.523 |
| Reproductive System Disease | 1.86E-07 | -1.071 |
| Skeletal and Muscular Disorders | 1.87E-07 | 2.199 |
| Cellular Function and Maintenance | 2.16E-07 | -0.271 |
| Cellular Assembly and Organization,Tissue Development | 2.21E-07 | -0.333 |
| Cancer,Reproductive System | 2.82E-07 | 0.053 |
| Cancer,Respiratory Disease | 3.27E-07 | 0.279 |
| Cancer,Hematological Disease | 3.34E-07 | -0.132 |
| Cancer,Gastrointestinal Disease,Hepatic System Disease | 3.41E-07 | -0.518 |
| Cancer,Hepatic System Disease | 3.68E-07 | 0.779 |
| Cellular Motion | 3.75E-07 | 0.304 |
| Infectious Diseases, virus | 3.87E-07 | 0.523 |
| Immunological Disease,Organismal Injury and Abnormalities | 4.24E-07 | 0.239 |
| Dermatological Diseases and Conditions | 4.73E-07 | -0.152 |
| Hereditary Disorder,Neurological Disease | 4.79E-07 | 0.053 |
| Gastrointestinal Disease,,Organismal Injury and Abnormalities | 4.63E-04 | 0.278 |

Top cell function, associated with membrane SFPQ bound proteins,

identified by Ingenuity Pathway Analysis.

**Supplemental Table 3** Top 50 Canonical Pathway SFPQ binding protein involved

| Ingenuity Canonical Pathways | -log(p-value) | z-score |
| --- | --- | --- |
| EIF2 Signaling | 1.46E+01 | -2.714 |
| Oxidative Phosphorylation | 8.17E+00 | 1.312 |
| Cell Cycle Control of Chromosome Regulation | 5.87E+00 | 0.342 |
| BMP signaling pathway | 5.14E+00 | -2 |
| Purine Nucleotides De Novo Biosynthesis II | 4.47E+00 | 0.816 |
| Necroptosis Signaling Pathway | 3.97E+00 | 0.447 |
| Mitotic Roles of Polo-Like Kinase | 3.86E+00 | 0.345 |
| TCA Cycle II | 3.55E+00 | 0.251 |
| Insulin Secretion Signaling | 2.94E+00 | -0.23 |
| Apoptosis Signaling | 2.66E+00 | 0.816 |
| Coronavirus Pathogenesis signaling | 2.65E+00 | -0.352 |
| ILK Signaling | 2.64E+00 | -0.742 |
| BAG2 Signaling Pathway | 2.59E+00 | 0.322 |
| RAN Signaling | 2.46E+00 | 0.45 |
| Estrogen Receptor Signaling | 2.33E+00 | -0.421 |
| Androgen Signaling | 2.19E+00 | -0.342 |
| HIPPO signaling | 2.19E+00 | -0.276 |
| TGF-β Signaling | 2.09E+00 | -0.653 |
| Sirtuin Signaling Pathway | 2.09E+00 | 0.542 |
| mTOR Signaling | 2.08E+00 | -0.126 |
| ERK5 Signaling | 2.03E+00 | 0.432 |
| Coronavirus Replication Pathway | 1.87E+00 | 0.657 |
| Senescence Pathway | 1.84E+00 | -0.236 |
| cholecystokinin/gastrin-mediated signaling | 1.79E+00 | 0.213 |
| AMPK Signaling | 1.79E+00 | -0.215 |
| Glucocorticoid Receptor Signaling | 1.79E+00 | 0.436 |
| Neuregulin Signaling | 1.79E+00 | 2 |
| tRNA Charging | 1.67E+00 | -0.761 |
| Unfolded protein response | 1.67E+00 | 2 |
| Actin Cytoskeleton Signaling | 1.66E+00 | 0.543 |
| Fatty Acid Î²-oxidation I | 1.66E+00 | 0.548 |
| Folate Transformations I | 1.64E+00 | 0.451 |
| Cdc42 Signaling | 1.63E+00 | NaN |
| RhoA Signaling | 1.61E+00 | 0.349 |
| NRF2-mediated Oxidative Stress Response | 1.61E+00 | NaN |
| Isoleucine Degradation I | 1.61E+00 | NaN |
| Superpathway of Citrulline Metabolism | 1.60E+00 | NaN |
| Acyl Carrier Protein Metabolism | 1.52E+00 | NaN |
| Lanosterol Biosynthesis | 1.51E+00 | 1.342 |
| PAK Signaling | 1.49E+00 | NaN |
| Valine Degradation I | 1.49E+00 | NaN |
| Huntington's Disease Signaling | 1.49E+00 | NaN |
| PI3K/AKT Signaling | 1.49E+00 | 0.341 |
| Regulation of Cellular Mechanics by Calpain Protease | 1.49E+00 | NaN |
| Tumoricidal Function of Hepatic Natural Killer Cells | 1.47E+00 | NaN |
| Glycine Biosynthesis I | 1.45E+00 | NaN |
| Ephrin Receptor Signaling | 1.41E+00 | NaN |
| Clathrin-mediated Endocytosis Signaling | 1.40E+00 | NaN |
| ERK/MAPK Signaling | 1.40E+00 | -0.217 |
| p70S6K Signaling | 1.40E+00 | NaN |

Top canonical pathways, associated with membrane SFPQ bound proteins,

identified by Ingenuity Pathway Analysis.

**Table 4: Primary antibodies and their working concentrations**

Antibody Ig G Isotype/Source/Cat. No./Clone Concentration

SFPQ Mouse IgG/Proteintech/67129-1-AP/Monoclonal 1:1000

E cadherin Mouse IgG/Millipore/3199z/Monoclonal 1:1000

Ki67 Mouse IgG/ R&D /NB500-170/Monoclonal 1~2µg/ml

MMP2 Rabbit IgG/ Abcam / ab97779 / rabbit monoclonal 1:1000

PPIA Rabbit IgG/ Abcam / ab97779 / rabbit polyclonal 1:1000

Lamin A Rabbit IgG/ Abcam / ab108595/ rabbit monoclonal 1:800

VIM Rat IgG/ EMD Millipore/ MAB3400/ rat monoclonal 1:500

S100A4 Rabbit IgG/ Abcam/ ab124805// rabbit monoclonal 1:500

GAPDH Mouse IgG/Thermo Sci/ MA1-16757/MAab 1:1000

H2AX Rabbit IgG/ abcam / ab11175/polyclonal 1:1000
